# TREATMENT OF GASTRIC CANCER ACCORDING TO THE COMPLEXITY OF THE HOSPITAL ONCOLOGY UNIT: ANALYSIS OF 33,774 PATIENTS OVER TWO DECADES

**DOI:** 10.1590/0102-6720202400052e1846

**Published:** 2024-12-16

**Authors:** Marcus Fernando Kodama Pertille RAMOS, Marina Alessandra PEREIRA, Carolina Terra de Moraes LUIZAGA, Valeria LOMBARDO, Valter Bezerra LEITE, Stela Verzinhasse PERES, Rodrigo Nascimento PINHEIRO, Ulysses RIBEIRO

**Affiliations:** 1 Universidade de São Paulo, Faculty of Medicine, Hospital das Clinicas, Cancer Institute, Department of Gastroenterology – São Paulo (SP), Brazil;; 2 Fundação Oncocentro de São Paulo – São Paulo (SP), Brazil;; 3 Sociedade Brasileira de Cirurgia Oncológica – São Paulo (SP), Brazil.

**Keywords:** Centralized Hospital Services, Cancer Care Facilities, Stomach Neoplasms, Gastrectomy, Survival Analysis, Oncology Service, Hospital, Serviços Centralizados no Hospital, Institutos de Câncer, Neoplasias Gástricas, Gastrectomia, Análise de Sobrevida, Serviço Hospitalar de Oncologia

## Abstract

**BACKGROUND:**

The hospitals’ volume, specialization, availability of all oncological services, and experience in performing complex surgeries have a favorable impact on gastric cancer (GC) treatment.

**AIMS:**

The aim of this study was to compare the results of GC treatment according to the type of oncological hospital in the State of São Paulo.

**METHODS:**

Patients diagnosed with GC between 2000 and 2022 in qualified hospitals for cancer treatment were evaluated by data extracted from the hospital cancer registry. Patients were assessed according to the type of hospital for cancer treatment: Oncology High Complexity Assistance Unit (UNACON) and Oncology High Complexity Care Center (CACON), which has greater complexity.

**RESULTS:**

Among the 33,774 patients, 23,387 (69.2%) were treated at CACONs and 10,387 (30.8%) in UNACONs. CACON patients were younger, had a higher level of education, and had a more advanced cTNM stage compared to UNACON (all p<0.001, p<0.05). The time from diagnosis to treatment was over 60 days in 49.8% of CACON’s patients and 39.4% of UNACON’s (p<0.001, p<0.05). Surgical treatment was performed in 18,314 (54.2%) patients. The frequency pN0 (40.3 vs 32.4%) and pTNM stage I (23 vs 19.5%) were higher in CACON. There was no difference in overall survival (OS) between all adenocarcinoma cases treated at CACON and UNACON (9.3 vs 10.3 months, p=0.462, p>0.05). However, considering only patients who underwent curative surgery, the OS of patients treated at CACON was better (24.4 vs 18 months, p<0.001).

**CONCLUSIONS:**

Patients with GC who underwent gastrectomy at CACONs had better survival outcomes, suggesting that the centralization of complex cancer surgery may be beneficial.

## RESUMO

**RACIONAL:**

O volume do Hospital, especialização, disponibilidade serviços oncológicos e experiência na realização de cirurgias complexas têm impacto favorável no tratamento do câncer gástrico (CG).

**OBJETIVOS:**

Comparar resultados do tratamento do CG de acordo com o tipo de Hospital oncológico no Estado de São Paulo.

**MÉTODOS:**

Pacientes diagnosticados com CG entre 2000 e 2022 foram avaliados por dados extraídos do Registro Hospitalar de Câncer, de acordo com o tipo de Unidade de tratamento: Unidade de Assistência de Alta Complexidade Oncológica (UNACON) e Centro de Assistência de Alta Complexidade Oncológica (CACON), que tem maior complexidade.

**RESULTADOS:**

Entre os 33.774 pacientes, 23.387 (69,2%) foram tratados em CACONs e 10.387 (30,8%) em UNACONs. Pacientes no CACON eram mais jovens, tinham maior nível de escolaridade e cTNM mais avançado em comparação ao UNACON (todos p<0,001). O tempo do diagnóstico ao tratamento foi superior a 60 dias em 49,8% dos pacientes do CACON e 39,4% dos do UNACON (p<0,001). O tratamento cirúrgico foi realizado em 18.314 (54,2%) pacientes. A frequência de pN0 (40,3 vs 32,4%) e pTNM I (23 vs 19,5%) foi maior no CACON. Não houve diferença na sobrevida global (SG) entre todos os casos de adenocarcinoma tratados no CACON e no UNACON (9,3 vs 10,3 meses, p=0,462, p>0.05). No entanto, considerando apenas os pacientes submetidos à cirurgia, a SG daqueles tratados no CACON foi melhor (24,4 vs 18 meses, p<0,001, p<0.05).

**CONCLUSÕES:**

Pacientes com GC submetidos à gastrectomia em CACONs apresentaram melhores resultados de sobrevida, sugerindo que a centralização de cirurgias oncológicas complexas pode ser benéfica.

## INTRODUCTION

The burden of neoplasms as a cause of morbimortality has increased worldwide. In this context, the creation of hospitals qualified for specialized and comprehensive care for cancer patients has been occurring throughout the world^14^. Among the most relevant tumors, gastric cancer (GC) is still the fifth most common and third most lethal in the world^3,23^. Surgical resection remains the main therapeutic modality, and, currently, the combination of perioperative chemotherapy (CMT) has a proven role in improving survival^1,10^. In this way, the treatment of GC in specialized cancer hospitals has the benefit of better surgical expertise, in addition to straightforward access to CMT^2^.

According to Brazil’s National Cancer Prevention and Control Policy from 2013, patients must receive specialized and comprehensive assistance, so that early detection, diagnosis, staging, treatment, rehabilitation, and palliative care are offered promptly, allowing continuity of care. Hospitals authorized by the Ministry of Health to treat cancer patients are defined as High Complexity Care Units (UNACON), High Complexity Oncology Care Centers (CACON), and general hospitals with surgical oncology services^5,7^.

UNACONs are hospital units that have adequate technical conditions, physical facilities, equipment, and human resources to provide specialized assistance for the definitive diagnosis and treatment of the most prevalent cancers. It must include both surgical and medical oncology services. They may have in their physical structure or be able to refer patients to other units to receive radiotherapy, hematological, and pediatric assistance if necessary^5,7^.

CACONs are hospital units that have the same resources as UNACONs and must be able to treat all types of cancer, but not necessarily rare and childhood cancers. They must necessarily have radiotherapy and hematology services, as well as surgical and clinical oncology services that must also be available at UNACONs. Although both types of units are qualified for the treatment of cancer, CACONs are considered more specialized cancer centers and perform a larger number of surgical procedures^3,5,7^.

The relationship between hospital specialization and results for complex oncological surgeries such as GC is already well established^2^. Thus, the inverse relationship between hospital volume and mortality persists, where it is estimated that low-volume hospitals may have a surgical mortality rate up to four times higher for complex surgeries^6^. The beneficial effect of specialized care is justified by standardized clinical guidelines, experienced multidisciplinary teams, and the availability of sufficient resources such as intensive care units and interventional radiology.

Therefore, we aimed to compare the results of GC treatment in UNACONs and CACONs in the State of Sao Paulo.

## METHODS

All patients included in the hospital cancer registry maintained by the Fundação Oncocentro de São Paulo (FOSP) database with ICD C16 for gastric neoplasms from January 2000 to February 2022 were considered eligible. FOSP is a public institution created in 1974 to encourage research, teaching, and assistance in oncology, stimulating activities for the prevention and early detection of cancer. In the State of São Paulo, FOSP is also responsible for the coordination, restructuring, and processing of the cancer registry at the state level.

Patients who had already undergone some previous cancer treatment, non-adenocarcinoma histological types, and treatment carried out in general hospitals that were not designated as UNACONs or CACONs were excluded.

Clinical data available included sex, age, educational level, and tumor location. The treatment variables included surgery, radiotherapy, and chemotherapy. Survival was evaluated according to the pTNM staging and type of hospital unit — UNACON or CACON.

The local ethics committee of the Hospital das Clinicas — University of São Paulo Medical School approved this study, and it was registered online (plataformabrasil.saude.gov.br; CAAE: 60549522500000068). Informed consent of patients was waived due to the retrospective design of the study. FOSP participated in the study as a co-participating institution, based on the Technical Cooperation Agreement, which provides for the availability of databases and guidance on their use, by the general law of data protection 13.709/18 (process 001.08003.000096/2020). The use of the database of the Hospital Cancer Registry of the State of São Paulo (RHC/SP) and the letter of consent for authorization of data with sensitive information were signed. All the necessary precautions were taken to secure the privacy of human subjects in the database, allowing the medical records and database to be used only by the investigators.

### Statistical analysis

Data were expressed as mean (with standard deviation, SD±) or median (with interquartile range) for continuous variables and as numbers with percentages for categorical data. Continuous and categorical variables were compared between the two groups using the standard t-test and chi-square test, respectively. Survival curves were assessed using the Kaplan-Meier method and compared using the log-rank test. Overall survival (OS) was the duration between the date of diagnosis or surgical resection (for operated patients) to death or last follow-up. Multivariate analysis to identify the independent prognostic factors was performed using the Cox proportional hazard regression model. All statistical tests were two-sided, and p<0.05 were considered significant. Statistical analyses were carried out using SPSS software, version 20 (SPSS, Chicago, IL).

## RESULTS

**Figure 1:**
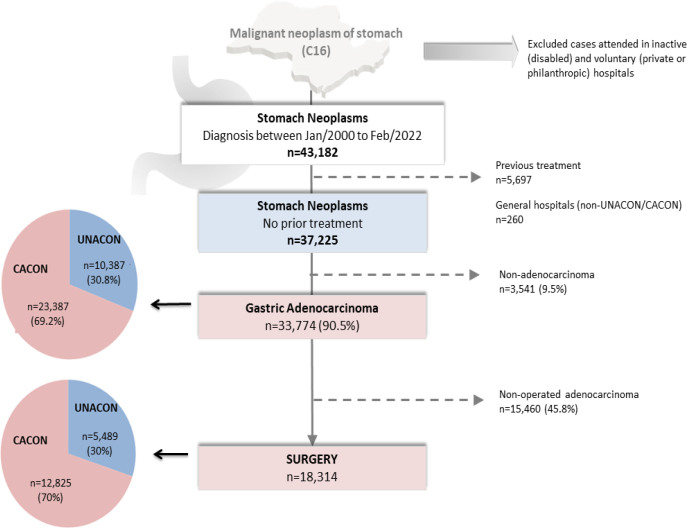
Study flowchart.

During the study period, 43,182 patients were initially selected. After applying the exclusion criteria, 33,774 patients diagnosed with adenocarcinoma remained for analysis. Among the 33,774 patients, 23,387 (69.2%) were treated at 15 CACONs and 10,387 (30.8%) were treated at 51 UNACONs. Surgical treatment was performed in 18,314 (54.2%) patients. The study’s flowchart is shown in Figure 1.

Regarding clinical characteristics of patients treated at UNACONs and CACONs, we found that patients in UNACON were younger (63.5 vs 62.8, p<0.001), and patients in CACON had a higher percentage of university degree (5.4% vs 3.5%, p>0.001) (Table 1). Tumors without a definition of location and histological type were more common in the UNACON group (59.7 vs 48% and 66.8% vs 46.1%, respectively). Previous diagnosis of the tumor was more frequent in the CACON group, meaning that the patients had the diagnosis in other institutions and were then referred. The time from diagnosis to treatment was over 60 days in 49.8% of CACON patients and 39.4% of UNACON (p<0.001). Surgery and radiotherapy were more commonly performed at CACONs and chemotherapy at UNACONs.

**Table 1:** Clinical characteristics and treatment modality of all patients with gastric cancer who underwent treatment at UNACONs and CACONs.

Variables	UNACON	CACON	p-value
	n=10,387 (%)	n=23,387 (%)	
Sex			
Female	3,511 (33.8)	7,966 (34.1)	0.642
Male	6,876 (66.2)	15,421 (65.9)	
Age (years)			
Mean (SD)	63.5 (13.0)	62.8 (13.3)	<0.001
Educational level			
Illiterate/incomplete elementary school	4,052 (58.8)	11,473 (62.3)	<0.001
Complete elementary/high school	2,596 (37.7)	5,933 (32.2)	
University	240 (3.5)	997 (5.4)	
Tumor location			
Antrum/pylorus	2,215 (21.3)	5,605 (24)	<0.001
Body	805 (7.8)	2,510 (10.7)	
Cardia/fundus	748 (7.2)	3,128 (13.4)	
Curvatures	416 (4.0)	909 (3.9)	
Others	6,203 (59.7)	11,235 (48)	
Histological type			
Intestinal	2,041 (16.9)	6,262 (26.8)	na
Diffuse/mixed	1,261 (12.1)	6,141 (26.3)	
Undifferentiated/other types	148 (1.4)	209 (0.9)	
Adenocarcinoma (unspecified)	6,937 (66.8)	10,775 (46.1)	
cTNM			
I	1,370 (13.9)	2,748 (13.1)	<0.001
II	1,433 (14.5)	2,715 (12.9)	
III	2,536 (25.7)	4,990 (23.8)	
IV	4,531 (45.9)	10,533 (50.2)	
Previous diagnosis			
No	5,199 (50.1)	7,642 (32.7)	<0.001
Yes	5,188 (49.9)	15,745 (67.3)	
Days – Diagnosis to treatment			
Median (IQR)	48 (24–84)	60 (33–98)	0.005
<60 days	4,177 (60.6)	8,355 (50.2)	<0.001
>60 days	2,718 (39.4)	8,300 (49.8)	
Surgery			
No	4,898 (47.2)	10,562 (45.2)	0.001
Yes	5,489 (52.8)	12,825 (54.8)	
Radiotherapy			
No	8,990 (86.6)	19,201 (82.1)	<0.001
Yes	1,397 (13.4)	4,186 (17.9)	
Chemotherapy			
No	4,955 (47.7)	11,865 (50.7)	<0.001
Yes	5,432 (52.3)	11,522 (49.3)	

UNACON: High Complexity Care Units; CACON: High Complexity Oncology Care Centers; SD: standard deviation.

The evaluation of only the patients who underwent surgical treatment did not demonstrate any differences in the sex and age of both groups (Table 2). We found a higher proportion of patients with the pN0 category (40.3% vs 32.4%, p<0.001) and pTNM stage I (23% vs 19.5%, p=0.002) in the CACON group.

**Table 2:** Clinicopathological characteristics of patients who underwent surgical treatment at UNACONs and CACONs.

Variables*	UNACON	CACON	p-value
	n=5,489 (%)	n=12,825 (%)	
Sex			
Female	1,915 (34.9)	4,550 (35.5)	0.444
Male	3,574 (65.1)	8,275 (64.5)	
Age (years)			
Mean (SD)	62.3 (12.6)	61.9 (12.8)	0.053
pT			
pT0/Tis/T1	424 (13.8)	979 (16.4)	0.002
pT2	529 (17.2)	990 (16.5)	
pT3	1,470 (47.9)	2,679 (44.8)	
pT4	648 (21.1)	1,336 (22.3)	
pN			
pN0	970 (32.4)	2,345 (40.3)	<0.001
pN+	2,021 (67.6)	3,474 (59.7)	
pN			
pN0	970 (32.4)	2,345 (40.3)	<0.001
pN1	904 (30.2)	1,526 (26.2)	
pN2	761 (25.4)	1,272 (21.9)	
pN3	356 (11.9)	676 (11.6)	
pM			
pM0	1,941 (77.6)	4,259 (80.1)	0.010
pM1	561 (17.8)	1,057 (17)	
pTNM			
I	613 (19.5)	1,431 (23)	0.002
II	1,091 (34.7)	2,082 (33.5)	
III	878 (27.9)	1,643 (26.4)	
IV	561 (17.8)	1,057 (17)	

*missing data in some patients; UNACON: High Complexity Care Units; CACON: High Complexity Oncology Care Centers; SD: standard deviation.

### Survival analysis

**Figure 2:**
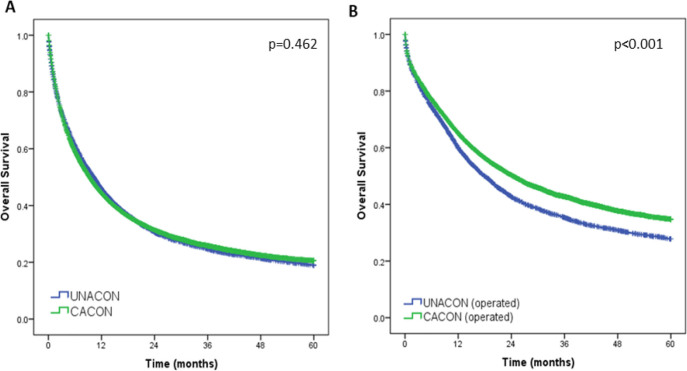
Overall survival according to UNACON and CACON. (A) All patients and (B) only patients who underwent surgery.

UNACON: Oncology High Complexity Assistance Unit; CACON: Oncology High Complexity Care Center.

During follow-up, 74.9% of patients died. The mean follow-up time for all patients was 18 months, and the mean follow-up time for living patients was 42.2 months (median of 60 months). Regarding all patients, there was no difference in OS between CACON and UNACON (median OS: 9.3 vs 10.3 months, p=0.462). However, when evaluating only the groups of patients who underwent surgery, patients treated at CACON had better survival compared to UNACON (median OS: 24.4 vs 18 months, p<0.001) (Figure 2).

**Figure 3:**
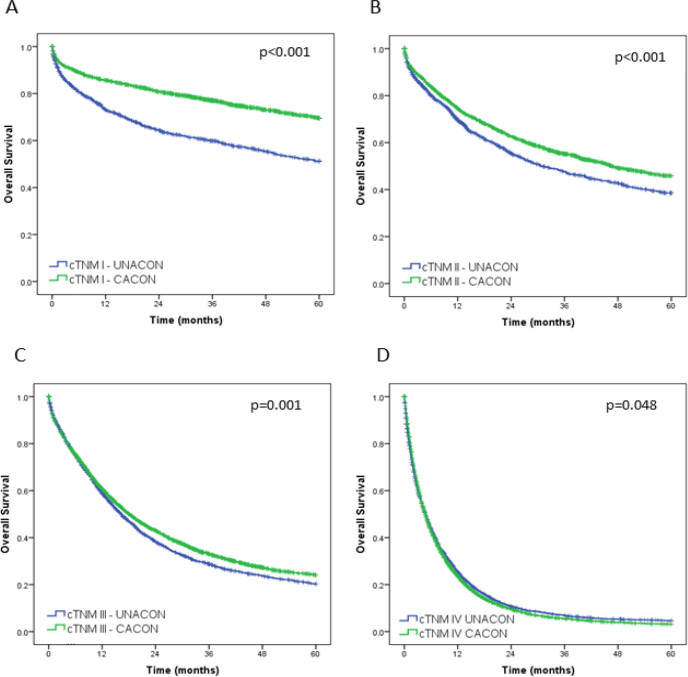
Overall survival for UNACON and CACON according to TNM clinical stage.

UNACON: Oncology High Complexity Assistance Unit; CACON: Oncology High Complexity Care Center.

The survival curves of CACON and UNACON groups according to the cTNM stage are shown in Figure 3. Patients treated at CACONs had better survival than UNACONs in stages I, (median not reached, p<0.001), II (median 31 vs 46.6 months, p<0.001), and III (median 16.3 vs 18.1 months, p<0.001). However, patients with clinical stage IV treated at UNACONs showed better survival than CACONs (median 4.8 vs 4.7 months, p=0.048).

In the analysis of factors associated with survival in patients who underwent surgery, age = 65 years, male sex, advanced pTNM categories, and surgical treatment performed at UNACONs (HR=1.17, 95%CI 1.10–1.24, p<0.001) were associated with worse OS (Table 3).

**Table 3:** Univariate and multivariate analysis of variables associated with survival of patients who underwent surgical treatment.

Overall survival	Univariate		p-value	Multivariate		p-value
Variables	HR	95%CI		HR	95%CI	
Male (vs female)	1.26	1.21–1.31	<0.001	1.16	1.08–1.23	<0.001
Age =65 (vs <65 years)	1.27	1.22–1.32	<0.001	1.41	1.33–1.50	<0.001
pT1 (reference)						
vs pT2	2.34	2.04–2.68	<0.001	1.88	1.61–2.19	<0.001
vs pT3	4.22	3.74–4.75	<0.001	2.90	2.52–3.34	<0.001
vs pT4	7.55	6.67–8.55	<0.001	4.16	3.59–4.81	<0.001
pN0 (reference)						
vs pN1	1.94	1.80–2.09	<0.001	1.39	1.28–1.51	<0.001
vs pN2	2.80	2.60–3.01	<0.001	1.74	1.59–1.89	<0.001
vs pN3	3.25	2.97–3.55	<0.001	1.75	1.58–1.93	<0.001
pM1 (vs pM0)	3.47	3.26–3.70	<0.001	2.10	1.95–2.27	<0.001
UNACON (vs CACON)	1.20	1.16–1.25	<0.001	1.17	1.10–1.24	<0.001

HR: Hazard ratio; CI: Confidence interval; UNACON: High Complexity Care Units; CACON: High Complexity Oncology Care Centers.

## DISCUSSION

In the Brazilian National Unified Health System (SUS), cancer patients are initially treated at the Basic Health Unit (UBS) or in a General Hospital. Once the cancer diagnosis has been established, patients are then referred to UNACON or CACON authorized by the Ministry of Health for treatment. The organization of patient care flow is the responsibility of the State and Municipal Health Departments. The general recommendation is that the chosen referral oncology service should be located closest to the patient’s residence, as treatment can often be long^3,5^. The State of São Paulo has an estimated population of around 44 million inhabitants, and its territory is divided into 17 Regional Health Care Networks. Only two regional networks have neither UNACON nor CACON.

According to Federal Law 12,732 of 2012, patients diagnosed with cancer must begin treatment within 60 days of diagnosis^19^. In both groups, only about half the patients started treatment during this period, demonstrating the need for greater agility in referral. This delay was greater in the CACON group, which was expected given that there are fewer institutions in this category.

Another result that may have influenced this delay in CACONs is that tumor diagnosis was performed more frequently at UNACONs. It should be noted that neither type of institution is necessarily dedicated exclusively to the treatment of cancer. Thus, UNACONs, as they are less complex hospitals, have a profile more similar to a General Hospital and may diagnose cancer when investigating a patient’s symptoms. CACON, being a more specialized center, ends up receiving the majority of patients with a previous diagnosis.

The level of education can be used as an indicator of the patient’s socioeconomic status^13^. One of the negative aspects of case centralization is the possibility of excluding patients with more precarious economic conditions from the possibility of treatment. The impossibility of traveling long distances and the economic impact of absence from work affect this group of vulnerable patients the most^17^. As there are fewer CACONs compared to UNCAONs, it is clear that to reach a CACON a greater displacement is necessary. Indeed, we found a higher frequency of patients with a university degree in the CACON group, suggesting a possible exclusion of patients with disadvantageous socioeconomic conditions^7^.

The main therapeutic modality for GC remains surgical resection, but the addition of perioperative chemotherapy to the treatment has been increasingly indicated. In this context, treating patients in specialized centers facilitates the coordination of different specialties. So, according to this, we found a greater frequency of multidisciplinary treatment in CACONs.

In survival analysis between both groups, we found no difference when all patients were evaluated. It should be remembered that almost half of the patients evaluated had clinical stage IV. In this group of patients, the main treatment modality is palliative systemic chemotherapy^21,22^. The availability of drugs for chemotherapy in the public health system is the same for both CACONs and UNACONs, a fact that may justify the similar results between the groups. The OS of clinical stage IV was even better at UNACONs.

On the other hand, when only cases undergoing surgery were evaluated, survival was significantly higher in the CACON group. Gastrectomy with adequate lymphadenectomy has already been highlighted as one of the surgeries that benefit from case centralization in specialized high-volume centers^15,18,21,24^. Hospitals specializing in the treatment of complex cancer patients develop standardized protocols for perioperative care and management of complications. Not only the surgical team but also the nursing team is more attentive to the earlier identification of complications. Critical resources, such as ICU capacity and interventional radiology support, are more readily available in hospitals that care for a high volume of complex patients^2^. This leads to an increase in the possibility of rescuing patients who present complications, having an important impact on survival^8,15,16,21^.

Unfortunately, centralizing complex surgeries in high-volume specialized centers is not always possible due to barriers including socioeconomic disparities, geographic constraints, and patient preference^4,9,11,12,20^. A point of criticism of the centralization of cases would be that the results may simply reflect a selection bias of the most favorable cases, which end up being sent and treated in referral centers^14^. Another long-term deleterious effect of centralization is to make non-specialized hospitals increasingly less able to treat cancer patients. As many cancer patients still occasionally need to seek care in non-specialized hospitals, these situations end up being managed less efficiently.

As a strength of our study, we highlight the wide coverage of the population treated in the State of São Paulo. Both UNACONs and CACONs must maintain a functioning hospital cancer registry, linked to FOSP, for the systematic and continuous collection of cancer cases treated in the institution. This guarantees universal data collection, but unfortunately, patients who are treated outside the cancer care network in general or private hospitals are not included in this registry. Therefore, we were unable to compare the results of treatment carried out outside the specialized cancer treatment network. Another limitation is related to the lack of details of the surgical treatment. The extent of gastric resection and mainly the type of lymphadenectomy involved are important quality and prognostic parameters. Perioperative morbidity and mortality and pathological outcomes, such as the number of dissected lymph nodes and resection margin, are also parameters that could be useful when comparing groups.

In this study, we were able to evaluate the profile of patients treated for GC in the State of São Paulo in the last 20 years. Unfortunately, there were a large number of clinical stage IV patients, a fact that serves as a warning for the need to increase early diagnostics and adoption of screening programs in high-risk populations. The better survival of patients operated on in CACONs suggests the benefit of case centralization in specialized centers.

## CONCLUSIONS

Patients with GC who underwent surgical treatment at CACONs, a more specialized hospital, had better survival outcomes than those at UNACONs. These results suggested that the centralization of complex cancer surgery for GC patients may achieve better results if referred to high-volume specialized centers.
